# Cilostazol attenuates the severity of peripheral arterial occlusive disease in patients with type 2 diabetes: the role of plasma soluble receptor for advanced glycation end-products

**DOI:** 10.1007/s12020-015-0545-6

**Published:** 2015-02-11

**Authors:** Jhih-Syuan Liu, Tsung-Ju Chuang, Jui-Hung Chen, Chien-Hsing Lee, Chang-Hsun Hsieh, Tsung-Kun Lin, Fone-Ching Hsiao, Yi-Jen Hung

**Affiliations:** 1 Division of Endocrinology and Metabolism, Department of Internal Medicine, Tri-Service General Hospital, National Defense Medical Center, #325, Sec. 2, Chen-Kung Rd., Nei-Hu, Taipei, Taiwan; 2 Graduate Institute of Medical Sciences, National Defense Medical Center, Taipei, Taiwan; 3 Medical Supplies and Maintenance Office, Tri-Service General Hospital, National Defense Medical Center, Taipei, Taiwan

**Keywords:** Cilostazol, Peripheral arterial occlusion disease, Soluble receptor for advanced glycation end product

## Abstract

Recent studies have demonstrated that the plasma soluble receptor for advanced glycation end-products (sRAGE) play a major role in developing macrovascular complications of type 2 diabetes, including peripheral arterial occlusion disease (PAOD). Cilostazol is an antiplatelet, antithrombotic agent, which has been used for the treatment of PAOD. We hypothesized that cilostazol attenuates the severity of PAOD in patients with type 2 diabetes through the augmentation of plasma sRAGE. Ninety type 2 diabetic patients with PAOD defined as intermittent claudication with ankle-brachial index (ABI) ≦0.9 were recruited for an open-labeled, placebo-controlled study for 52 weeks with oral cilostazol 100 mg twice daily (*n* = 45) or placebo (*n* = 45). Fasting plasma sRAGE, endothelial variables of E-selectin, soluble vascular cell adhesion molecule-1 (sVCAM-1), and inflammatory markers of high-sensitivity C-reactive protein (hsCRP) and tumor necrosis factor-α (TNF-α) were determined. After completely the 52-week treatment program, the ABI values were elevated in cilostazol group (*P* < 0.001). The plasma sRAGE was significantly increased (*P* = 0.007), and hsCRP, sVCAM, and E-selectin concentrations were significantly decreased (*P* = 0.028, <0.001 and <0.001, respectively) with cilostazol treatment. In a partial correlation analysis with adjustments for sex and age, the net change of sRAGE significantly correlated with the change of ABI in the cilostazol group (*P* = 0.043). In a stepwise multiple regression model, only the change with regards to sRAGE was significantly associated with the change of ABI (*P* = 0.046). Our results suggest that cilostazol may effectively attenuate the severity of PAOD in patients with type 2 diabetes. Plasma sRAGE plays a role as an independent predictor for improving the index of PAOD.

## Introduction

The prevalence of type 2 diabetes is rising at an alarming rate worldwide and has become a leading cause of morbidity and mortality associated with accelerated atherosclerosis, including coronary artery, cerebrovascular, and peripheral arterial occlusive disease (PAOD) [1]. Recent studies demonstrate that PAOD correlates strongly with the risk of major cardiovascular events and has a high prevalence of coexistent coronary and cerebrovascular disease, especially in type 2 diabetes [2]. Because the prevalence of PAOD also increases progressively with age, PAOD is a growing clinical problem due to the increasingly aged population worldwide [3].

In addition to the established cardiovascular risk factors, recent studies reported reduced plasma circulating levels of receptor for advanced glycation end-products (sRAGE) as an important novel biomarker in hypertension, type 2 diabetes, vascular dementia, and in non-diabetic subjects with coronary artery disease [4–9]. One recent study also demonstrated that patients combined with coronary artery disease and PAOD have lower sRAGE levels than patients with coronary artery disease alone [10]. RAGE has a C-truncated secreted isoform, termed soluble RAGE (sRAGE). RAGE is a multiligand receptor that engages diverse ligands relevant to the atherogenesis of atherosclerosis [11]. However, vascular endothelial cells exposed to a variety of RAGE ligands augments the RAGE activation to result in enhanced generation of reactive oxygen species and in the activation of the transcription factor and nuclear factor kB (NF-kB). This leads to sustained upregulation of proinflammatory mediators, adhesion molecules, and to an initiation of atherosclerosis [12].

Cilostazol is an antiplatelet, antithrombotic agent that has been used for the treatment of chronic PAOD and for the secondary prevention of brain infarction [13]. Cilostazol not only inhibits platelet activation but also increases vasodilatation [14]. In addition, it has been shown to inhibit vascular smooth muscle cell proliferation, then improve peripheral blood flow and insulin sensitivities via attenuation of inflammation process [15, 16]. Given that the anti-atherogenic effect of cilostazol is ascribed to its property to suppress superoxide and TNF-α formation, and thereby reduces NF-kB activation and transcription, vascular cell adhesion molecule/monocyte chemotactic protein-1 (VCAM/MCP-1) expressions, and monocyte recruitments in low-density lipoprotein (LDL) receptor-null mice [17]; it remains unclear as to whether cilostazol improves the severity of PAOD via the pathway of RAGE to influence downstream signals such as inflammation and adhesion molecules [18]. Accordingly, the purpose of this study was designed as an open, placebo-controlled study evaluating cilostazol in patients with type 2 diabetes plus PAOD. We will test the hypothesis that cilostazol may ameliorate the severity of PAOD in patients with type 2 diabetes via the augmentation of plasma circulating RAGE.

## Materials and methods

### Subjects and study design

An open, placebo-controlled study was conducted. The study protocol was approved by institutional review boards. All subjects provided written informed consent to participate in the study prior to the initiation of the study. The study enrolled subjects 35–80 years of age with type 2 diabetes that had been first diagnosed with this disease after being 30 years of age. Patients with type 2 diabetes plus PAOD were selected to participate in this study to evaluate the ankle-brachial index (ABI), inflammation markers, and subjective improvement effects using oral cilostazol 100 mg twice daily or placebo for the 52-week treatment period. PAOD was documented with ABI of ≦0.9 in the reference leg when the pressures are taken by pulses value record in a supine position. Other inclusion criteria were as follows: type 2 diabetes with A1c 7.0–12 % with stable medication condition prior to 3 months and dyslipidemia or hypertension with stable medication condition prior to 3 months.

Intermittent claudication was ascertained using a standardized physician-administered questionnaire. The specific questions were leg discomfort on exertion that was related to ground steepness or rapidity of walking that was relieved with rest. Participants were questioned with regards to cigarette smoking habit at each examination.

Subjects were excluded from the study if they had type 1 diabetes mellitus; females of childbearing potential (Patients who have been surgically sterilized bilateral tubal ligation or hysterectomy or who are at least 1 year post-menopausal may participate in the study) or females of lactating; patients who had a history of heart failure, myocardial infraction, coronary vascular disease, or unstable angina pectoris within recent 6 months; chronic kidney failure (on dialysis any kind, kidney implantation); any history within the previous year of clinically significant bleeding tendencies, hemorrhagic tendencies; malignancy of any kind; use of an investigational drug within the past 3 months; had impaired liver function (aspartate aminotransferase and/or alanine aminotransferase >2 times the upper limit of reference range); or had any uncontrolled or untreated systemic disease considered by the investigator to make them unfit to enter the study.

Ninety eligible subjects were enrolled and visited the investigator four times with 12-week intervals for further efficacy and safety assessment, and the time schedule was set on the following date during the study. The primary efficacy end point was changes with regards to ABI and sRAGE after 52-week treatment. The secondary efficacy parameters were the changes in A1c, blood pressure, and lipid profile concentrations over the treatment period from baseline to the end of the study. In addition, changes in plasma inflammatory markers of TNF-α and hsCRP, endothelial markers of E-selectin, soluble intercellular adhesion molecule-1 (sICAM-1), and sVCAM-1 were also measured.

### Laboratory measurements

After maintaining a 10 h fasting state, blood samples were obtained from each participant to determine plasma glucose, A1c, creatinine, and lipid profiles. Plasma circulating hsCRP, tumor TNF-α, E-selectin, sICAM-1, and sVCAM-1 were subsequently measured. Serum total cholesterol, triglyceride, and low-density lipoprotein cholesterol (LDL-C) were measured using the dry, multilayer analytical slide method in the Fuji Dri-Chem 3000 analyzer (Fuji Photo Film Corporation, Tokyo, Japan). The levels of A1c were evaluated by ion-exchange high-pressure liquid chromatography (HPLC) method (BIO-RAD VARIANT II, Los Angeles, CA, USA). Plasma glucose concentrations were determined by the glucose oxidase method on a Beckman Glucose Analyzer II (Beckman Instruments, Fullerton, CA, USA).

Plasma hsCRP levels were measured using the Tina-quant (Latex) high-sensitivity assay (Roche, Mannheim, Germany). The intra-assay and inter-assay CV for hsCRP were 3.7 and 4.9 %, respectively. Serum TNF-α was measured with the Biotrak™ high-sensitivity human ELISA kit from Amersham Biosciences (Buckinghamshire, UK). The intra-assay and inter-assay CV for TNF-α were 3.5 and 5.3 %, respectively. Levels of E-selectin, sICAM-1, and sVCAM-1 were measured by commercial ELISA (R&D Systems, Minneapolis, USA). The intra-assay and inter-assay CV for E-selectin were 4.5 and 6.2 %; sICAM-1 were 3.5 and 7.1 %; sVCAM-1 were 5.0 and 8.7 %.

Serum sRAGE levels were determined using ELISA (Quantikine; R & D systems, Minneapolis, MN, USA) according to the manufacturer’s protocol. Briefly, a monoclonal antibody raised against the N-terminal extracellular domain of human RAGE consisting of amino acids Gln 24 through Ala 344 was used to capture sRAGE from serum. Captured sRAGE was detected with a polyclonal antihuman sRAGE antibody. After washing, plates were incubated with streptavidin horseradish peroxidase, developed with appropriate substrate and OD450 was determined using an ELISA plate reader. The intra- and inter-assay CV were 2.1 and 5.9 %, respectively. All of the concentrations for the above biochemical variables were determined in duplicate and the values of the two samples were averaged.

### Measurement of ABI

The ABI measurement of our study has been descripted previously [18]. Briefly, measurement of upper- and lower-extremity blood pressure was performed in a supine position after at least 30 min of rest. The blood pressure cuffs were placed over each brachial artery and above each malleolus. To calculate the ABI ratio, the average systolic blood pressure measurement in the ankle was divided by the average systolic blood pressure measurement in the arm. The mean pressure of the higher arm was used to calculate the ABI separately for each leg. An ABI greater than 1.5 was excluded.

### Statistical analysis

Arithmetic means and standard deviations (SD) were calculated for the variables measured at least on an interval scale. Efficacy end points of this study include ABI and sRAGE after treatment, subjective improvement scale, and inflammation or endothelial markers. Categorical variables were analyzed by the *χ* ^2^test. Continuous variables were tested for normal distribution by the Kolmogorov–Smirnov test. Statistically significant differences between the basal and treatment values within the groups were analyzed with Student’s *t* test. Mann–Whitney *U* test was tested for non-parametric method if necessary. Partial correlation analysis adjusting for age and sex was used to assess the correlation between the change of ABI and other variables on cilostazol treatment. Stepwise multiple linear regression analysis, with the changes of ABI as dependent variables, was used to study the association and independent determinants of covariates. A *P* value of less than 0.05 was considered to be statistically significant. All statistical analyses were performed using SPSS Inc. 16.0 software (SPSS, Chicago, IL, USA). The number of patients reporting side effects during the different treatments was recorded. In general, the incidence of side effects was low, and only a descriptive method was used.

## Results

**Fig. 1:**
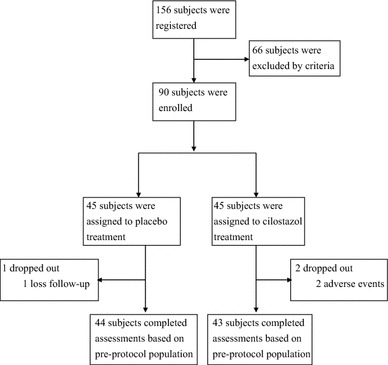
Flow diagram of our study

Of the 156 subjects from the Division of Endocrinology and Metabolism of the Tri-Service General Hospital in Taipei, Taiwan, assessed for eligibility, 90 subjects satisfied the inclusion criteria and were assigned to treatment with 1:1 randomization (Fig. 1). Forty-four of 45 subjects in the placebo group and 43 of 45 subjects in the cilostazol group took the study medication for at least 52 weeks with a compliance rate of >80 %. Due to an adverse event of severe dizziness, two subjects in the cilostazol group were withdrawn from treatment. In addition, one subject in the placebo group was lost to follow-up. No occurrence of serious adverse events, including death, danger to life, disability, or hospitalization (initial or prolonged) requiring intervention to prevent permanent impairment or damage, was observed during the study period.

There were no significant differences between both groups related to demographic and baseline characteristics as detailed in Table 1. After the completion of the 52-week treatment period, the ABI value was significantly elevated (0.84 ± 0.09 vs 0.89 ± 0.11, *P* < 0.001) and HbA1c level was reduced (8.1 ± 1.3 vs 7.6 ± 1.4 %, *P* = 0.002) in the cilostazol group without significant alteration in the placebo group. However, increases in total cholesterol, LDL-cholesterol, and creatinine levels were demonstrated in the placebo group, but these were not found in the cilostazol group. No differences after treatment in terms of body mass index, blood pressure, fasting glucose, and triglyceride were observed in both groups.

**Table 1:** Baseline demographic data and clinical characteristics of the study groups

	Placebo (*n* = 44)	Cilostazol (*n* = 43)	*P* value
Age (year)	65.6 ± 7.8	67.1 ± 9.3	NS
Sex (M %)	30.2	36.2	NS
Body mass index (kg/m^2^)			
Before	26.8 ± 4.4	27.3 ± 4.5	NS
After	26.6 ± 4.4	27.0 ± 4.5	
*P* value	NS	NS	
Systolic pressure (mmHg)			
Before	135.4 ± 18.0	138.0 ± 14.3	NS
After	132.7 ± 17.7	137.2 ± 12.8	
*P* value	NS	NS	
Diastolic pressure (mmHg)			
Before	80.5 ± 9.0	80.4 ± 8.7	NS
After	80.1 ± 9.5	80.9 ± 7.3	
*P* value	NS	NS	
Fasting glucose (mg/dl)			
Before	141.7 ± 29.6	139.7 ± 44.8	NS
After	155.0 ± 57.6	138.4 ± 48.5	
*P* value	NS	NS	
HbA1c (%)			
Before	8.3 ± 1.1	8.1 ± 1.3	NS
After	8.1 ± 1.0	7.6 ± 1.4	
*P* value	NS	**0.002**	
Total Cholesterol (mg/dl)			
Before	182.1 ± 35.7	177.7 ± 33.1	NS
After	192.5 ± 35.8	186.4 ± 41.0	
*P* value	**0.022**	NS	
LDL-C (mg/dl)			
Before	109.6 ± 28.1	108.4 ± 33.6	NS
After	115.9 ± 28.0	114.5 ± 30.3	
*P* value	**0.046**	NS	
Triglyceride (mg/dl)			
Before	153.6 ± 69.4	161.7 ± 74.4	NS
After	161.0 ± 82.3	157.1 ± 91.0	
*P* value	NS	NS	
Creatinine (mg/dl)			
Before	1.02 ± 0.31	0.96 ± 0.33	NS
After	1.11 ± 0.35	1.01 ± 0.41	
*P* value	**0.009**	NS	
ABI (affected limb)			
Before	0.84 ± 0.06	0.84 ± 0.09	NS
After	0.83 ± 0.07	0.89 ± 0.11	
*P* value	NS	**<0.001**	

Bold values indicate statistical significance

**Fig. 2:**
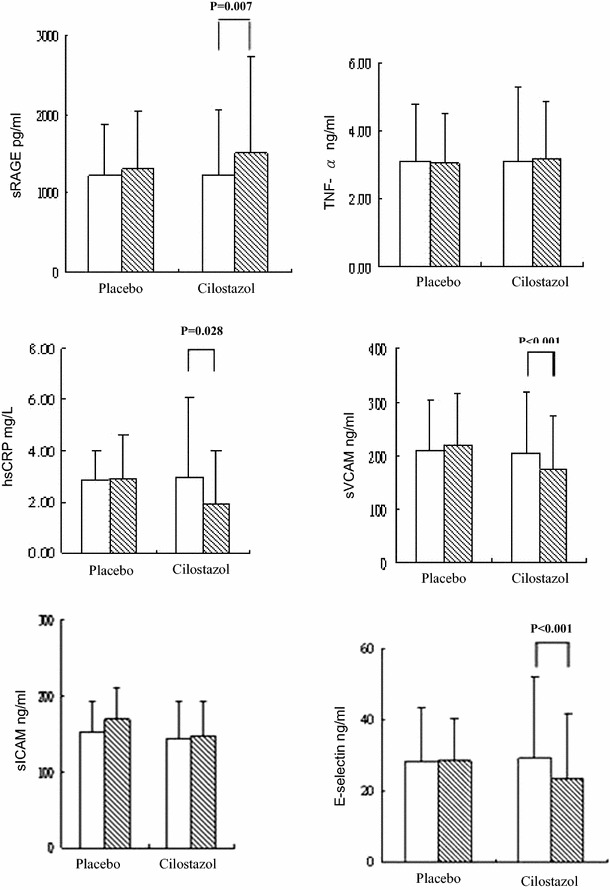
Comparing with placebo, levels of plasma sRAGE were significantly increased and hsCRP, sVCAM and E-selectin concentrations were significantly decreased at the end of 52 weeks with cilostazol treatment. Data were expressed as mean ± SD. Statistical analysis was conducted with the Mann–Whitney *U* test. *sRAGE* soluble receptor for advanced glycation end-products, *TNF-α* tumor necrosis factor-α, *hsCRP* high-sensitive C-reactive protein, *sICAM-1* soluble intercellular adhesion molecule-1, and *sVCAM-1* soluble vascular cell adhesion molecule-1

The levels of plasma sRAGE were significantly increased (1234.1 ± 822.5 vs 1510.0 ± 1215.7 pg/ml, *P* = 0.007) and hsCRP (3.0 ± 3.2 vs 1.9 ± 2.1 mg/L, *P* = 0.028), sVCAM (205.1 ± 113.7 vs 172.7 ± 101.2 ng/ml, *P* < 0.001), and E-selectin concentrations (29.1 ± 23.0 vs 23.4 ± 18.5 ng/ml, *P* < 0.001) were significantly decreased with cilostazol treatment in Fig. 2. No statistically significant differences in terms of TNF-α and sICAM were shown after treatment in both groups. In Spearman partial correlation analysis with adjusting for sex and age, only net changes of sRAGE were significantly correlated with changes of ABI in cilostazol group (*r* = 0.385, *P* = 0.043) (Table 2). By stepwise multiple regression analysis, only changes of sRAGE was determined to be an independent predictor of changes of ABI (*β* = 0.367, *P* = 0.046) as documented in Table 3.

**Table 2:** Spearman partial correlation coefficients between the changes of ankle-brachial index and the differences in variables with regards to cilostazol treatment

Variables	*r*	*P*
BMI	0.246	0.207
SBP	0.018	0.927
DBP	0.323	0.093
Fasting glucose	−0.127	0.518
HbA1c	0.160	0.416
LDL-C	−0.116	0.556
Total cholesterol	−0.148	0.454
Triglyceride	−0.291	0.134
Creatinine	0.154	0.435
sRAGE	0.385	**0.043**
TNF-α	0.047	0.813
hsCRP	−0.129	0.513
sVCAM	−0.217	0.266
sICAM	0.182	0.354
E-selectin	−0.211	0.282

Corrected for age and sex. Bold value indicates statistical significance

**Table 3:** Stepwise multiple linear regression analysis with the change of ankle-brachial index as the dependent variable

	Unstandardized coefficients		Standardized coefficients		
Variables	*B*	SE	*β*	*t*	*P* value
Constant	0.051	0.015		3.412	0.002
sRAGE	5.012E−5	0.001	0.367	2.090	0.046

## Discussion

The influence of plasma AGE/sRAGE levels, inflammation markers, and adhesion molecules on cilostazol therapy, particularly in humans, remains unclear. The main finding of present study demonstrates that cilostazol effectively improves the severity of PAOD, defined as the index of ABI in patients with type 2 diabetes via an augmentation of plasma circulating sRAGE and an attenuation of proinflammatory markers, subsequently affecting the adhesion molecules regulation. The enhancement of plasma sRAGE plays a role as an independent determinant for improving the severity of peripheral arterial insufficiency.

Cilostazol, a phosphodiesterase III (PDE III) inhibitor, decreases the activity of PDE III, and then accumulation of 3′-5′ cyclic adenosine monophosphate (cAMP) levels occurred in the platelets, smooth muscle cells, as well as decreases intracellular calcium which leading to cellular relaxation. Cilostazol has antiplatelet effects, has a vasodilator effect on smooth muscle cells and anti-proliferative activity on smooth muscle cells [2]. Moreover, Shichijo M et al. have shown that cilostazol suppresses cytokine production in a mastocyte culture [19]. It also blocks the production and expression of MCP-1 induced by TNF-α, and inhibits VCAM-1 via suppression of the nuclear transcription factor kappa B (NF-kB) in culture of endothelial cells taken from a human umbilical cord [20]. The ligand of AGEs binding to the receptor of AGEs activates the inflammatory process leading to an over-expression of downstream signals such as VCAM-1, ICAM-1, and E-selectin on cultured human endothelial cells [21]. This is combined with a higher expression of the mRNA for the respective adhesion molecules [22]. This result implicates that the combination of matrix glycation and inflammation up-regulates the activation of the endothelial cell adhesion cascade. Exposure of human endothelial cells to glycated albumin leads to an increase in human monocyte adhesion to endothelium and an induction of both soluble and cell-associated expression of VCAM-1, ICAM-1, and E-selectin. There was also an augmentation in the levels of these molecule transcripts and an increase in the DNA binding activity for NF-kB in the promoters of these antigens observed [23].

Animal studies also demonstrated that sRAGE have been proved to be benefit in decreasing atherosclerosis in diabetic mice [24, 25]. Recent study has revealed negative correlations between levels of sRAGE and coronary heart disease in diabetes [26]. Hence, we speculated that cilostazol inhibits the signaling of NF-kB and decreases transcription of different proteins, including ICAM-1, VCAM-1, E-selectin, TNF-α, interleukin-6 (IL-6), AGE, and ligands for RAGE in human atherosclerosis. Meanwhile, using in vitro studies (unpublished data), we provided evidence that cilostazol decreased RAGE, VCAM-1, and ICAM-1 expression and might be through inhibiting RAGE/ERK/NF-κB pathway. Less circulation sRAGE serves as a scavenger receptor for decreasing serum AGEs and other RAGE ligands. As a result, increasing concentrations of sRAGE, as well as decreasing levels of E-selectin and sVCAM may occur in diabetic subjects with cilostazol treatment [21, 27, 28]. Moreover, subsequent suppression of CRP production was also displayed via degradation of cytokines such as IL-6 and TNF-α in hepatocytes [29, 30].

Another clinical effect of cilostazol is the modification of lipid profiles, including reducing the concentrations of chylomicrons, remaining very low-density lipoprotein (VLDL), triglycerides as well as increasing HDL in patients with PAOD [31–35]. However, these beneficial effects of lipids were not observed significantly but only a mild improvement in our study. Besides, our study did not control for dyslipidemia, concomitant use of lipid-modifying agents, diet, poor diabetes control, or other factors that may influence serum lipids prospectively.

The metabolic parameters changed in both the cilostazol and placebo groups, especially decreased HbA1c level in cilostazol groups. Better glycemic control was found in the cilostazol group probably through the reduction of insulin resistance by reducing inflammation [16]. The same results were also observed in other animal studies [36, 37]. Although there were elevations observed in other lipid profile and chemical tests in the cilostazol group, namely total cholesterol, LDL-C, and creatinine, the increases were small and the results did not reach a level of statistical significance, including a comparison to the placebo group.

There were a number of limitations in this study that are worth highlighting. First, our study did not demonstrate a causal relationship between the severity of PAOD and circulating sRAGE, but only showed their association with one another. Second, ABI measurement only provides us with a simple tool to define the severity of peripheral arterial insufficiency. Angiography or magnetic resonance angiography may be more precise. Furthermore, non-invasive transcutaneous oxygen tension (TcPO2) had better predictive value than that of ABI, in predicting cardiovascular events and evaluating PAOD [38].

In summary, our results suggest that that cilostazol may effectively attenuate the severity of PAOD in patients with type 2 diabetes. Plasma sRAGE plays a role as an independent determinant for improving the index of peripheral arterial insufficiency.
